# Beyond DNA binding - a review of the potential mechanisms mediating quinacrine's therapeutic activities in parasitic infections, inflammation, and cancers

**DOI:** 10.1186/1478-811X-9-13

**Published:** 2011-05-15

**Authors:** Reza Ehsanian, Carter Van Waes, Stephan M Feller

**Affiliations:** 1 Tumor Biology Section, Head and Neck Surgery Branch, National Institute on Deafness and Other Communication Disorders, National Institutes of Health, Bethesda, MD, USA; 2 Stanford University School of Medicine, Stanford, CA, USA; 3 Cell Signalling Group, Department of Molecular Oncology, Weatherall Institute of Molecular Medicine, John Radcliffe Hospital, Oxford University, Headley Way, Oxford OX3 9DS, UK

## Abstract

This is an in-depth review of the history of quinacrine as well as its pharmacokinetic properties and established record of safety as an FDA-approved drug. The potential uses of quinacrine as an anti-cancer agent are discussed with particular attention to its actions on nuclear proteins, the arachidonic acid pathway, and multi-drug resistance, as well as its actions on signaling proteins in the cytoplasm. In particular, quinacrine's role on the NF-κB, p53, and AKT pathways are summarized.

## Nomenclature and chemical grouping

**Figure 1:**
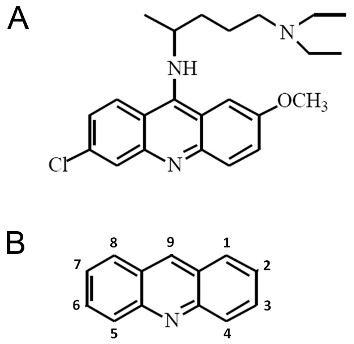
**Structures of acridine and the acridine family member quinacrine**. (A) Chemical structure of the acridine family member quinacrine. (B) Chemical structure of acridine.

Quinacrine (IUPAC name 4-N-(6-chloro-2-methoxyacridin-9-yl)-1-N,1-N-diethylpentane-1,4-diamine) is a heterocyclic three-ring compound (Figure 1A), and an acridine (Figure 1B) derivative (9-aminoacridine). It is readily available as quinacrine dihydrochloride, the dihydrochloride salt of quinacrine, for clinical use. The interest in quinacrine stems from its long history of therapeutic uses, as will be discussed in the following sections, and in particular its potential antineoplastic activities.

Quinacrine formulations and isomers are known by numerous designations some of which are: acrichine, Atabrine^®^, atebrine, atebrin, mepacrine, quinacrine dihydrochloride, quinacrine dihydrochloride dihydrate, quinacrine dihyrochloride (R)-isomer, quinacrine dihyrochloride (S)-isomer, quinacrine dimesylate, quinacrine hydrochloride, quinacrine monoacetate, quinacrine monohydrochloride, quinacrine monomesylate, quinacrine (R)-isomer, quinacrine (S)-isomer, and 6-chloro-9-[[4-(diethylamino)-1 methylbutyl]amino]-2-methoxyacridine. The most commonly used designations for quinacrine are mepacrine, quinacrine hydrochloride, quinacrine dihydrochloride, and the registered name Atabrine^®^. Quinacrine is one of several known aminoacridines which include, for example, acridine orange, acriflavine, aminacrin, amsacrine, ethacridine, nitracrine, proflavine and tacrine and which have a range of biological and therapeutic applications. Table 1 summarizes some of the key biological and therapeutic applications of these compounds.

**Table 1:** Selected aminoacridines and their typical applications

Acridine orange	A cationic cytochemical stain specific for cell nuclei, especially DNA. It is used as a supravital stain and in fluorescence cytochemistry. It may cause mutations in microorganisms.
Acriflavine	3,6-Diamino-10-methylacridinium chloride mixture. with 3,6-acridinediamine. Fluorescent dye used as a local antiseptic and also as a biological stain. It intercalates into nucleic acids thereby inhibiting bacterial and viral replication.

Aminacrine	A highly fluorescent anti-infective dye used clinically as a topical antiseptic and experimentally as a mutagen, due to its interaction with DNA. It is also used as an intracellular pH indicator.

Amsacrine	Aminoacridine derivative that is a potent intercalating antineoplastic agent. It is effective in the treatment of acute leukemias and malignant lymphomas, but has poor activity in the treatment of solid tumors. It is frequently used in combination with other antineoplastic agents in chemotherapy protocols. It produces consistent but acceptable myelosuppression and cardiotoxic effects.

Ethacridine	A topically applied anti-infective agent.

Nitracrine	Acridine antineoplastic agent used in mammary and ovarian tumors. It inhibits RNA synthesis.

Proflavine	3,6-Diaminoacridine. Topical antiseptic used mainly in wound dressings.

Tacrine	A cholinesterase inhibitor that crosses the blood-brain barrier. Tacrine has been used to counter the effects of muscle relaxants, as a respiratory stimulant, and in the treatment of Alzheimer's disease and other central nervous system disorders.

Quinacrine	An acridine derivative formerly widely used as an antimalarial but superseded by chloroquine in recent years. It has also been used as an anthelmintic and in the treatment of giardiasis and malignant effusions as well as a form of contraception/sterilization. It is used in cell biological experiments as an inhibitor of phospholipase A2.

Modified from National Library of Medicine - Medical Subject Headings; 2009 MeSH; MeSH Descriptor Data. http://www.nlm.nih.gov/cgi/mesh/2009/MB_cgi?mode=&term=Aminoacridines&field=entry#TreeD03.494.046.250; retrieved July 2010.

## History of quinacrine

Originally developed as pigments and dyes, the pharmalogical properties of acridine compounds were first investigated by Ehrlich and Benda in 1912, as antiprotozoal agents that act upon trypanosome parasites and developed further by Carl Browning as antibacterial agents [1-3]. The use of acridines as antibacterial agents fell out of favor in the 1940's after the discovery and wide spread availability of penicillin to combat bacterial infections. However, from the 1940's to the present day acridines have found wide use as antimalarial agents with Atabrine^® ^(quinacrine) being one of the acridine derivatives successfully used to combat the disease. Atabrine^® ^was discovered as part of an intensive antimicrobial research program broadly based on biologically active dyes carried out in 1930's in the German laboratories of I.G. Farbenindustrie. The program covered the preparation and trial of over 12,000 compounds leading to the identification of pamaquine and quinacrine as potential therapeutic agents [4].

Quinacrine was re-discovered in American laboratories as "American Atabrine" during the Second World War when an alternative to quinine was needed for the treatment of malaria [4]. The outcomes from the use of quinacrine in the armed forces demonstrated it to be superior to quinine and made it the official medicine for the treatment of malaria [5] until 1945 when it was substituted by chloroquine [6]. Before the substitution, millions of military personal took Atabrine^® ^for prophylaxis. This allowed physicians in the US armed forces to conduct extensive follow-up studies and provide health professionals with detailed information on the side effects and toxicity of quinacrine, making it among the best studied drugs ever introduced. Three million soldiers took the drug for up to four years in the controlled setting of the military service where arguably compliance and follow up rates are much better than in a typical study in the civilian population [7-9].

Throughout the years, the use of quinacrine has continued attaining FDA-approval for the treatment of diseases such as malaria, giardiasis [10-13] and tapeworm infection [14-16]. Its therapeutic effectiveness has also been demonstrated in controlled studies in combating refractory lupus erythematosus [17-22], rheumatoid arthritis [21,23], and as an adjuvant cancer therapy [24,25]. In addition, quinacrine has been used as an intrapleural sclerosing agent to prevent recurrence of pleural effusion or pneumothorax in patients at high risk of recurrence, resulting in painless pleurodesis and remission of fluid and/or air collections [26-30]. Quinacrine has also been used for regional cancer therapy of pericardial and abdominal effusions with an ~25-50% responses rate [31]. Due to its effectiveness as a sclerosing agent, quinacrine has also been utilized for contraceptive purposes. It produces an asymptomatic fibrosis and occlusion of the fallopian canal [32-35]. It should be noted that for some of these conditions quinacrine has been superseded by other agents, however the use of quinacrine has to date not become contraindicated due to safety concerns. Quinacrine is currently being clinically tested in the treatment of Creutzfeldt-Jakob disease through the National Institute of Aging (NIA) [ClinicalTrials.gov Identifier: NCT00183092] and through the Medical Research Council, in the PRION-1 trial, [ClinicalTrials.gov Identifier: NCT00104663]. In addition, a trial has recently been completed in the treatment of androgen-independent prostate cancer through the University of Chicago and Cleveland Biolabs [ClinicalTrials.gov Identifier: NCT00417274].

## Pharmacokinetics of quinacrine

The typical route of quinacrine administration is orally with water after a meal [36]. The drug can also be administered intralesionally/paralesionally [21,25,37,38], intramuscularly, rectally, intravenously [21], transcervically [34], and interstitially [26-30,39].

It is rapidly absorbed from the gastrointestinal tract following oral administration [40] with plasma levels increasing 2-4 hours after administration and reaching a peak in 8-12 hours [7,21]. Plasma concentration increases rapidly during the first week and equilibrates (94%) by the fourth week. Quinacrine is also rapidly absorbed and distributed after intrapleural, intralesion/paralesion, and intrauterine administration [41,42]. The plasma levels of quinacrine remain low in comparison to tissue concentrations. Peak plasma concentrations of up to 140 ng/ml (0.32 μM) for quinacrine have been documented on a standard malaria regimen [8]. It is distributed throughout the body and its liberation from different tissue compartments is slow. The highest concentrations are found in the liver, spleen, lungs and adrenal glands, with liver concentrations reaching 20,000 times that of plasma. The lowest concentrations of the drug are found in the brain, heart and skeletal muscle [6,8]. Quinacrine is also heavily deposited in the skin, fingernails and hair [21]. Spinal fluid concentrations are 1-5% of plasma levels. 80-90% of the drug is bound to plasma proteins when given at therapeutic doses and the half life of the drug is five to fourteen days depending on the dosing regimen [41,43]. Although small amounts are excreted in bile, sweat, and saliva [21,40], the major route of quinacrine elimination is via the renal system which may be enhanced by acidification and reduced by alkalinization [6,7].

## Reported quinacrine toxicity

Quinacrine has the advantage of a long history of clinical use in the treatment of malaria, so that human tolerances are well known. In addition quinacrine has displayed tissue specificity making its toxicity tolerable in different therapeutic situations [21,44-46]. The following sections give an overview of the toxicity of quinacrine as it is applicable in the clinical setting.

## General toxicity

Mostly minor or reversible adverse reactions include transient symptoms of mild headache, dizziness, or gastrointestinal symptoms (diarrhea, anorexia, nausea, abdominal cramps) which decrease with a reduction in dosage [21]. These symptoms occur in half of the patient population receiving 100 mg of quinacrine daily while almost all patients treated with higher doses experience some sort of adverse reaction. Some infrequent serious side effects of quinacrine have been reported and will be covered in the following sections.

## Gastroenterological and hepatic toxicity

Persistent abdominal cramping or diarrhea has been reported for patients receiving the drug. These symptoms are readily dealt with by co-administration of bismuth-containing suspensions or antispasmodic agents. Long-term high-dose malarial suppressive therapy was occasionally associated with reversible hepatitis presumably due to quinacrine's tendency to concentrate in the liver. Transient lupus associated quinacrine hepatitis and peritonitis have also been reported, although these symptoms are attributed to doses three times that of the recommended dose [47,48].

## Ophthalmologic and central nervous system toxicity

Quinacrine has very low risk of retinal toxicity [49,50]. At doses over 500 mg the drug has the potential to induce in rare cases a hypersensitivity reaction resulting in corneal edema, which is reversible [51,52]. Cortical stimulatory effects of quinacrine were documented in a study of a group of healthy volunteers given doses of quinacrine ranging between 200 to 1,200 mg daily for ten days [53]. At higher doses symptoms may include restlessness, vertigo, insomnia, nightmares, hyperirritability, psychosis and convulsions. Although toxic psychosis following quinacrine administration has been reported [54-56], large scale studies reveal this to be a rare and quickly reversible event. However it must be noted that a study of over 7,500 US soldiers given quinacrine (100 mg/day) in World War II revealed a 0.4% incidence of toxic psychosis [57]. Further investigations revealed twenty eight (0.1%) CNS-toxic cases among 30,000 treated for malaria [58].

## Hematologic toxicity

The most serious potential toxicity of quinacrine is aplastic anemia. The incidence of aplastic anemia in World War II soldiers increased after the drugs introduction, but still remained quite low (0.003%) [59]. Reported cases of aplastic anemia have been associated with patients receiving more than the recommended daily dose and long treatment periods without having blood counts checked [60-65]. In considering this toxicity it is important to note that the potential lethality of aplastic anemia is readily preventable due to the early signs of skin rash. Moreover, hypoplastic anemia can be identified with frequent routine blood tests [60]. In the more modern clinical setting 300 mg/day of quinacrine has been administered and found to be reasonably tolerated with no reported incidence of hematologic toxicity [66].

## Dermatologic toxicity

In a study of 120,000 Australian soldiers serving during the Second World War only 1.6 percent developed rashes from quinacrine treatment. Eighty percent were eczematous and twenty percent were lichenoid or exfoliative [67]. Lichen planus was observed in 1 of 2,000 soldiers given 100 mg/day and in 1 of 500 given 200 mg/day. The dermatitis quickly resolved upon cessation of drug administration. Quinacrine can produce a yellow stain in the skin as well as areas of discoloration appearing like "black and blue marks" or bruises presumably due to melanin binding [67-69]. Slate-colored pigmentation of the palate and subungual areas were described in soldiers treated with quinacrine hydrochloride by Lippard and Kauer [69]. Hyperpigmentation of the oral mucosa, typically restricted to the hard palate has since been reported by many others [70]. These marks consist of membrane bound intracellular granules of quinacrine that contain large amounts of iron and some sulphur [67,71-76]. At the doses currently used, approximately half of the patients receiving the drug develop increased pigmentation and in half of these patients, an asymptomatic yellow stain is evident, which is reversible upon reduction to an average daily dose of <50 mg of the drug [21].

## Carcinogenicity/Tumorigenicity

There have been no studies conducted to investigate the tumorigenicity of orally administered quinacrine in humans. The data that exist document the use of quinacrine in female sterilization. Retrospective analysis revealed that patients administered with intrauterine quinacrine had a slight but not statistically significant increase in the incidence of cancer compared to a control population. However, the studies concluded that there was no evidence for an excess risk of cancer development [34,77-79].

Conflicting tumorigenicity data have been reported in short term (up to 30 days) animal studies. Studies in female mice and rats have shown that quinacrine (at doses of 30 mg/kg and 22.5 mg/kg, respectively) enhanced growth of implanted tumor cells and decreased survival [80,81]. However, other studies in male mice have shown that quinacrine at doses between 20-25 mg/kg suppress the growth of transplanted tumors and increase the rate of survival [82-84]. These inconsistencies may be explained by a recent study that reveals that dosing schemes in mice that are not equivalent to that used in humans lead to tumor formation [10]. While tumor formation in mice receiving dosages that are equivalent to those currently used in the clinic are equivalent to that of control animals [10].

## LD50 established in animal studies

The LD50 of quinacrine hydrochloride for rats is 900 mg/kg by oral administration [85,86]. The LD50 for the i.p. route for rats has not been estimated, but the experiments of Keeler and Richardson [87] suggest that it is approximately 250 mg/kg.

## Mechanisms of quinacrine as an anti-cancer agent

Most of the efforts in anti-cancer drug discovery have so far been focused on identifying drugs which target a single protein. Currently there is an increasing recognition for the need of rationally designed drugs that act on several different proteins and pathways [88-90]. Hence "polypharmacology", the term used for drugs that bind to and modulate multiple targets, thereby eliciting several clinical effects, is an exciting and developing area of cancer research [91]. The anti-cancer mechanism of quinacrine is complex, with many potential cellular targets. This "shotgun" nature of the drug is what may make it attractive in the treatment of some cancers. The following sections describe the different anti-cancer mechanisms elicited and signaling pathways modulated by quinacrine.

## Quinacrine intercalates into DNA

DNA is generally considered to be one of the biological targets for acridine anticancer compounds. There are three general modes of binding that characterize the compound interactions with double-stranded DNA: intercalation, groove binding and covalent binding [92-94]. Synthetic or natural acridine drugs display varying chemical and biological properties but they share the common property of DNA intercalation. This is due to the presence of an acridine "backbone" that confers a planar structure to the molecules, allowing them to intercalate into DNA by stacking between base pairs. The intercalation of several acridines has been demonstrated whereby the flat polyaromatic chromophore inserts between the base pairs of double-helical DNA. This process is driven by stacking and charge-transfer interactions between the aromatic systems of the acridine compounds and the DNA bases, resulting in unwinding of the helix [95,96]. The acridine derivative quinacrine is no exception, it also binds to DNA by intercalation [95,97-109]. It should be noted that intercalation is not the only type of interaction quinacrine has with DNA, another involves the diaminobutyl side chain which interacts with the minor groove of the DNA and is involved in the stabilization of the double helix against thermal strand separation [110,111].

Parameters such as fluorescence quantum yield (i.e. absorption/emission spectra), binding constant, and flexibility in the quinacrine/polynucleotides complexes have been found to strongly depend on the DNA sequence [102,112,113]. In general, a clear difference has been found between the fluorescence quenching of quinacrine when comparing adenine (A)-thymine (T) rich polynucleotides to guanine (G)-cytosine (C) containing ones. Fluorescence emission is enhanced in AT polymers, and a marked quenching observed in GC polymers [98,100,101,103,106-108,114]. Fluorescence-assayed preferential binding studies of quinacrine to DNA reveal that the neighbor base sequences influence the binding of quinacrine. In particular the sites where a GC base pair is involved were found to display high affinities [115]. The high affinity of quinacrine for DNA via intercalation can be hampered by denaturation or depurination [114,116].

## Acridine interaction with nuclear enzymes - potential mechanisms for anti-tumor effects

It has been demonstrated that DNA intercalation is necessary but not sufficient for the antitumor activities of acridines [109,117,118]. Although the chemotherapeutic potency of acridines is partly determined by the strength of DNA binding [117,119-121], the antitumor properties of acridines are not solely due to their DNA binding, but also stem from specific interactions with certain enzymes. Hence the toxicities of acridines are not largely due to an unspecific toxicity associated with DNA damage or binding.

## Telomerases as acridine targets

The two major classes of enzymes which have been considered as targets for these intercalating anticancer drugs are telomerases [122-126] and topoisomerases [127-131]. Topoisomerases have been well described as the target of many DNA-binding anti-cancer drugs while telomerases have more recently been the center of attention.

Telomerases are not active in normal somatic cells after birth. However, perhaps as many as 80-90% of cancer cells have reactivated telomerase [132]. Turning on this enzyme complex prevents or reverses telomere degradation and contributes to the growth of a malignant clone [133]. The inhibition of telomerase in cancer cells leads to growth arrest and ultimately cell death [132-134]. Acridines have been shown to help form or stabilize four-stranded intramolecular quadruplex structures (G-quadruplexes or G-quartets) from the guanine-rich DNA sequences of telomeres, which inhibit telomerase activity [123,126]. The formation of G-quadruplexes in telomeric DNA and the subsequent inhibition of telomerase make these conformations important as anti-cancer targets, and the drugs that help to form or stabilize them candidates for chemotherapeutic agents [135,136].

## Topoisomerases as acridine targets

Tumor cells are thought to over-express topoisomerase enzymes to enhance cellular proliferation. As the degree of topoisomerase poisoning and inhibition is a function of the amount of the enzyme present, this mechanism provides a potentially selective mode for killing of tumor cells [130,137]. By inhibiting the re-ligation activity of topoisomerase enzymes, acridines convert topoisomerases into DNA damaging agents leading to cellular toxicity and death [117,127-131].

## Quinacrine and telomerases

The mechanism of action of quinacrine on telomerase activity is not well described. Dominick et al. found that purified *E. aediculatus*, *T. thermophila*, and human telomerase was inhibited by quinacrine [138]. The banding patterns of the telomerase products generated in the presence of quinacrine were, however, not consistent with typical quadruplex stabilizing compounds which tend to cause enrichment of products associated with four repeats of the telomeric sequence [138,139]. Hence the exact process of quinacrine-induced inhibition of telomerase remains unclear. It should be noted that a 50 μM concentration of quinacrine was used to achieve telomerase inhibition, a dose well above the concentration needed to see the cytotoxic effects of quinacrine.

## Quinacrine and topoisomerase

Quinacrine is suggested to be a topoisomerase inhibitor as it displays intercalative activity and structural similarity to other acridines. Furthermore, quinacrine, like topoisomerase poisons, inhibits DNA repair [140-142]. It also inhibits excision repair processes in *E. coli *[143,144] and in human fibroblasts exposed to ultraviolet light [142,145-147]. In addition, quinacrine also sensitizes cultured HA1 cells (a sub-line of Chinese hamster ovary cells) to killing by X-rays and it prevents the repair of single strand breaks [148]. It has further been shown to sensitize cells when applied at the time of irradiation or shortly beforehand and to prevent the enzymatic rejoining of single strand breaks [148]. Hence it has been postulated that the observed radiosensitization is attributable to its capacity to inhibit such repair processes, in which topoisomerases are implicated [141,142,147,149]. It has also been hypothesized [142,146,147] that densely packed chromatin structures must be transiently loosened by topoisomerase to render the DNA damage sites accessible to excision repair enzymes, particularly for access of repair endonuleases that excise the damaged site [150-152].

It must be noted that the majority of reports which suggest that quinacrine is a topoisomerase inhibitor do not provide direct experimental evidence supporting the topoisomerase inhibitory activity of quinacrine. In one set of studies the effect of quinacrine on topoisomerase is assumed from experiments where quinacrine inhibits UV-induced DNA repair with a Kd of 38.1 μM for reparative DNA synthesis [142] and 781 μM for inhibition of DNA incision [146]. Hence in many studies the effect of quinacrine on topoisomerase is assumed to be due to the effect on DNA repair and when quinacrine is shown to inhibit this nuclear enzyme, the concentration required to induce this effect is quite high.

Although relatively few research reports exist that study the direct role of quinacrine in topoisomerase inhibition, a recent report revealed a lack of detectable topoisomerase interaction for quinacrine at doses up to 11 μM [109]. In another study, an indirect measure of topoisomerase activity, the P4 DNA unknotting assay, revealed that a concentration of 50 μM quinacrine was required to inhibit topoisomerase II P4 unknotting activity. However, in the same study the lowest IC_50 _for growth inhibition was attained in a cell line where drug resistance should have been encountered if the mechanism of action was due to topoisomerase inhibition [153]. Also in the same investigation, no DNA breakage and no DNA-protein binding was observed at lower doses of quinacrine which were observed to have an inhibitory effect on *in vitro *growth [153]. The notion that relatively high amounts of quinacrine are needed to interfere topoisomerase was shown by dose-dependent inhibition of topoisomerase enzyme activity, with 30-40% inhibition at 20 μM and 80-90% inhibition at 100 μM [141]. In addition, the high (>700 μM) concentration of quinacrine needed to induce DNA incision observed by Thielmann et al. [146] hints that enzymes involved in DNA repolymerization and not topoisomerase may be involved. Taken together these finding indeed support the role of other nuclear enzymes in the anti-tumor effect observed by quinacrine. From the body of evidence in the literature it is valid to assume that the stifled DNA repair observed with quinacrine is mediated by the inhibition of other enzymes, for instance repair-specific UV endonucleases, DNA helicases [154], or DNA polymerases [147], but not topoisomerases. One can also assume that at lower doses the effect of quinacrine may not be attributed to its interaction with the DNA and inhibition of nuclear enzymes as detailed further in later section of this review.

## Quinacrine effects on DNA and RNA polymerases

The literature describing the mechanism of quinacrine's anti-tumor effect suggests that two candidate families of nuclear enzymes, DNA polymerase and to a less extent RNA polymerase, may be involved in the mechanism of quinacrine's radiosensitizing ability. Effective nucleotide excision repair requires DNA gaps be filled by reparative DNA synthesis. In principle, all DNA polymerases found in the nucleus may play a role in this gap-filling. The effects of quinacrine on DNA and RNA polymerase reactions *in vitro *shed light on how quinacrine may inhibit enzymatic polymerization reactions *in vivo *and induce anti-tumor effects.

Early experiments have hinted at a mechanism of quinacrine preventing the action of DNA and RNA polymerase [155,156]. van Dyke et al. [155] demonstrated that quinacrine inhibits the incorporation of tritiated adenosine triphosphate primarily into RNA and DNA of the erythrocyte-free malaria parasite. In *Tetrahymena*, 32 μM quinacrine inhibits the synthesis of DNA (almost completely), RNA (70%), and protein (50%), and almost completely blocks the incorporation of labeled acetate into lipid components [156]. Evidence of quinacrine inhibition of DNA and RNA polymerase has also been obtained in *E. coli *K12, with RNA polymerase inhibition being less sensitive than DNA polymerase to quinacrine inhibition [110,111]. A K_d _in excess of 10 μM is reported by O'Brien *et al*. [110] and Hahn *et al*. [111]. More recent work revealed that when normal rat liver and Novikoff hepatoma DNA polymerases α, δ, and ε were treated with a dose range of 0.1 μM to 200 μM quinacrine, the drug preferentially inhibited the DNA polymerases from the malignant cells [157]. The IC_50 _values of quinacrine inhibition were 15.2 μM, 22.6 μM, and 11.4 μM for DNA polymerase α, δ, and ε, respectively, that were isolated from hepatoma, compared to that of 92.5, 200, and 146 μM for DNA polymerase α, δ, and ε isolated from normal rat liver [157]. The observed differences in DNA polymerase inhibition most likely reflects differences in the weakening effect on DNA-protein interactions [157], which in turn suggest a specific change in the DNA-binding domains of the individual polymerase enzymes. This hypothesis has been supported by the discovery of sequence changes of these DNA-binding domains for human and yeast DNA polymerases [158,159].

It should be noted that inhibition of DNA polymerases in other experiments is achieved at much higher concentrations of quinacrine. Inhibition of Hepatitis B virus DNA polymerase by quinacrine was only achieved at over 700 μM [160]. This agrees with the results of Thielmann *et al*. [146] where approximately the same concentration of quinacrine was needed to induce DNA incision in human fibroblasts. It should also be noted that using a different system to analyze the inhibitory effect of quinacrine on Hepatitis B virus DNA polymerase Hess et al. [161] found quinacrine only to be effective in the 20 to 50 mM range. Hence the cytotoxicity and anti-tumor effect of quinacrine achieved at lower dose well below those needed to generally inhibit polymerase activity must be attributed to other cellular mechanisms.

## Interaction with and inhibition of proteins involved in multidrug resistance

Multidrug resistance (MDR) is a major obstacle to the effective treatment of cancer, as MDR proteins aid in the active transport of a broad range of anticancer drugs out of the cancer cells. This export is ATP-dependent, allowing efflux against concentration gradients. An important set of proteins involved in this export is the ATP-binding cassette transporter family, which includes P-glycoprotein (P-gp). P-gp is encoded by the MDR1 gene and its overexpression is one of the major underlying mechanisms of MDR. The upregulation of P-gp in cancer cells has made it an attractive therapeutic target for combating MDR. One hypothesis supposes that P-gp allows cells to achieve MDR by actively pumping the chemotherapeutic agent out of the cells, thereby reducing the toxic effect [162,163]. The interaction of acridine-based chemotherapeutics with P-gp thus inhibits not only their own efflux but may also the efflux of co-administered chemotherapeutics, as well as increasing uptake into cells [164-168]. The interaction of acridine derivatives with proteins involved in MDR is not related to their DNA intercalation capabilities and appears to be an exciting new strategy for chemotherapy [162,167,169].

Quinacrine is implicated in the reversal of the MDR phenotype from several studies. It has been shown to reverse drug resistance to vincristine in a MDR sub-clone of K562 cells (a human chronic myelogenous leukemia cell line) starting at 5 μM [170]. Furthermore, it has been demonstrated to induce cytotoxicity, but the exact mechanism of cell death was not investigated [170]. The effect of quinacrine in reversing the MDR phenotype in leukemia cell lines *in vitro *was also supported by other investigators who used approximately 6 μM of quinacrine to increase cellular uptake of vincristine. They observed a cytotoxic effect with approximately 1aμM of quinacrine treatment, reducing cell growth by 82% when used alone, and almost completely inhibiting growth when combined with vincristine [171]. These same investigators then went on to conduct *in vivo *experiments showing the reversal of vincristine resistance with addition of 50 or 80 mg/kg/day of quinacrine [171].

The only direct test of the role of quinacrine as an inhibitor of P-gp has been conducted by using a multidrug resistant human T-cell leukemic cell line which expresses P-gp as a doublet that can be photaffinitty labeled by the analog of vinblastine, N(p-azido-[3-^125^I]salicyl)-N'-β- amlnoethylvindesine ([^125^I]NASV) [172]. [^125^I]NASV specifically binds to P-gp and the inhibition of its binding was used as a read out for the affinity of quinacrine for P-gp. In this study the binding affinity of quinacrine to P-gp was correlated to its ability to increase vinblastine sensitivity. It is, however, noteworthy that, although in an earlier investigation published by the same authors quinacrine increased the toxicity of vinblastine (12-fold) and vincristine (15-fold) at 5 μM and had an IC_50 _of 14 μM when administered alone [173], in this subsequent study a 50 μM dose only partially reduced [^125^I]NASV labeled P-gp [172].

The study of quinacrine's role in MDR has not been limited to leukemia but it has also been analyzed in MDR cells from the ovary and prostate cancer. Quinacrine was reported to affect MDR Chinese hamster ovary (CHO) cells at 6 μM in studies measuring the uptake of labeled palmitoyl carnitine and palmitoyl lysophosphatidyicholine. They were more rapidly taken up by the MDR cells and this uptake was reversed after quinacrine treatment back to the rates observed with the parental cell line, hence implicating quinacrine in reversing the MDR [174]. It also enhanced the activity of paclitaxel in hormone-refractory prostate cancer cells both *in vitro *and *in vivo *[175]. Quinacrine itself displayed IC_50 _values of 3.1 μM for PC-3, of 4.7 μM for PC-3M (a MDR sub-clone of the cell line) and of 3.5 μM for DU145 cells. Combination therapy of quinacrine and paclitaxel were determined to be synergistic in both, *in vitro *and *in vivo *(mouse xenografts) experiments. The exact mechanism of this synergistic effect was not studied however, the authors attributed to quinacrine's effect on phospholipase A_2 _(PLA_2_)[175].

## Disruption of the arachidonic acid pathway

**Figure 2:**
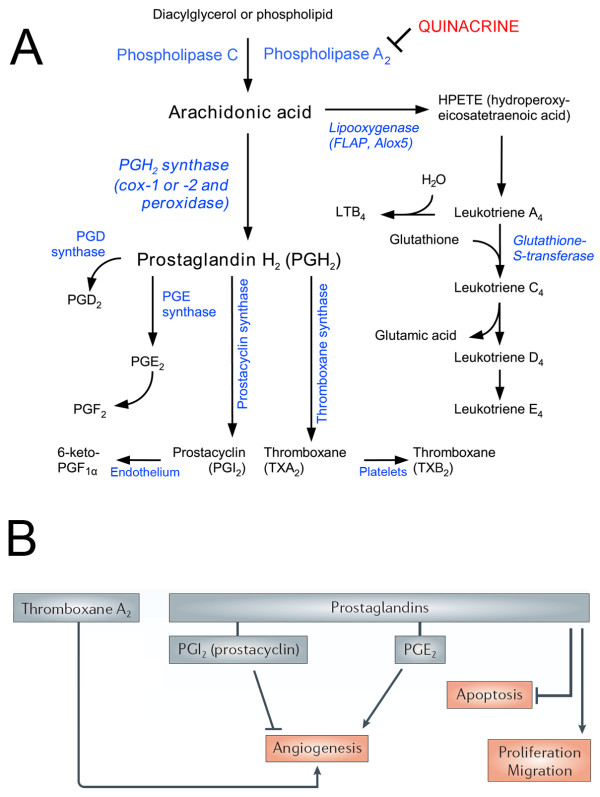
**The arachidonic acid pathway and its role in tumor promotion**. (A) Potential inhibitory role of quinacrine in the arachidonic acid pathway. (B) Potential role of arachidonic acid pathway in tumor promotion. Adapted from http://en.wikipedia.org/wiki/File:Eicosanoid_synthesis.png; retrieved January 2010 and Ulrich [278].

Manipulations of the arachidonic acid pathway (Figure 2) have received considerable attention in the chemoprevention of cancer [176-180]. Agents which inhibit this pathway have been demonstrated to hold promise in the chemoprevention of prostate, gastrointestinal, lung as well as esophageal cancer [177,181-184]. Although cyclooxygenase has been the focus of many anti-neoplastic agents targeting the arachidonic acid pathway [185], other components of the pathway could potentially also be promising targets. One such putative target, PLA_2_, hydrolyzes the sn-2-acyl bond of membrane phospholipids to produce arachidonic acid (Figure 2), which has been implicated in a variety of signal transduction events, including those regulating malignant cell proliferation [186,187]. Histological studies suggest that membrane phospholipase A_2 _expression levels are associated with tumor aggressiveness in gastric [188] and breast cancers [189].

Disruption of the arachidonic acid pathway by quinacrine via inhibition of PLA_2_, leads to a wide array of effects. The inhibition of PLA_2 _[190-193] occurs via quinacrine's binding to membrane phospholipids (primarily phosphatidylethanolamine), and subsequent intercalation into the membrane [194-198] and inhibition of PLA_2 _membrane binding and activity [190-193,199]. The decrease in arachidonic acid due to PLA_2 _inhibition [199] in turn results in the inhibition of leukotrienes (LOX activity) and prostanoids (COX activity), as well as eicosanoids (MOX/CYP450 activity) [193,194,200-210]. This is of interest since recent reports implicate an enhanced activity of arachidonic acid pathway proteins in preventing apoptosis and promoting tumor progression in head and neck cancer [211-222].

In platelets, the conversion of arachidonic acid to thromboxane is suppressed by quinacrine [21,206,210,223]. Thromboxane is a major factor in blocking the release of arachidonic acid from cellular phospholipases. In addition, thromboxane is involved in angiogenesis and the development of tumor metastasis [224-226]. Quinacrine also decreases prostaglandin E2 (PGE_2_) production in a dose-dependent manner. Prostaglandin, PGE_2 _and the COX2-PGE_2 _pathway/arachidonic acid pathway play an important role in the induction of the pro-inflammatory response and ultimately tumorigenesis [227-229]. PGE_2 _levels have been implicated in angiogenesis, tumor growth and invasion, apoptosis resistance and suppression of anti-tumor immunity via suppression of T and NK cells, and amplifying T_reg _[227,230-232]. The upregulation and pro-oncogenic actions of PGE_2 _have been demonstrated in head and neck cancer [222,233-237].

## Quinacrine as an inducer of p53 and inhibitor of the NF-κB and AKT pathways

**Figure 3:**
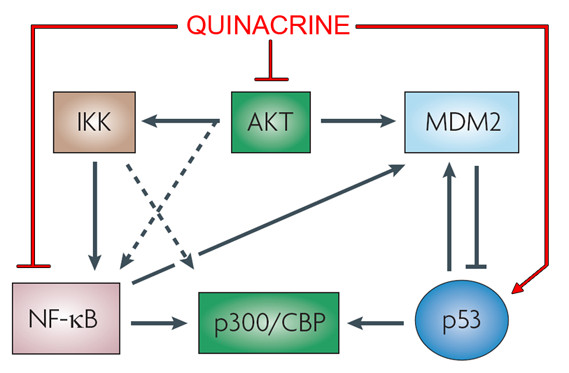
**Cross talk between NF-κB and p53**. There are many lines of crosstalk between the p53 and NF-κB pathways. A few of these are highlighted, such as AKT and the transcriptional co-activator proteins CREB-binding protein (CBP) and the related p300 protein. AKT can activate both IκB kinases (IKKs) and phosphorylate p65. AKT-mediated MDM2 phosphorylation can also inhibit p53 stabilization. Due to competition for the limited pool of CBP/p300, this protein also plays a crucial role in determining which pathway dominates in terms of cellular outcome. In addition, NF-κB has been shown to directly upregulate the levels of MDM2 mRNA and hence the protein. One promising aspect of quinacrine is its simultaneous ability to inhibit AKT, to induce the p53 pathway, and to inhibit the NF-κB pathway. Adapted from Dey et al. [279].

The deleterious roles of p53 inactivation [238,239] and nuclear factor-kB (NF-κB) hyperactivation [240,241] have been well established in human cancers. They lead to inhibition of cell death and promotion of oncogenesis. Cross talk between these two pathways has been identified and studied (Figure 3). It has been reported that p53 and NF-κB repress each other's activity by competing for transcriptional proteins such as p300 and CREB-binding protein (CBP) [242]. One signaling protein known to influence this competition is IKKα [243,244]. In particular, IKKα has been implicated in phosphorylating and directing CBP to participate in either the p53 or NF-κB pathway [243,244]. Another well studied signaling protein, AKT, can both activate IKKs as well as phosphorylate and enhance the transcriptional activity of p65 (NF-κB complex protein) [245-247]. In addition it has been demonstrated that AKT-mediated phosphorylation of MDM2 inhibits p53 stabilization [248]. Hence, it is likely that inhibitors of AKT activation could be utilized as anti-cancer agents for the inactivation of p53 and the inhibition NF-κB signaling.

Besides the development of AKT inhibitors, there has been no concerted effort to rationally design drugs that can simultaneously activate p53 and inhibit NF-κB. The opposing nature of these pathways suggests that a drug which activates p53 and simultaneously inhibits NF-κB would have significant clinical potential due to the fact that it is concomitantly modulating two critical cancer targets. In addition, a drug capable of affecting both of these pathways would also be a useful tool to study the interactions between the opposing p53 and NF-κB pathways. The literature [249] and further unpublished work from members of the Tumor Biology Group of NIDCD at NIH point to quinacrine as being such a drug (VanWaes et al., unpublished data).

## Quinacrine and p53

Several recent investigations support the notion that quinacrine-induced cell death is caused by a mechanism that is independent of DNA damage [250-252]. Quinacrine has been demonstrated to stabilize p53 in a manner that differs from that of DNA-damage induced p53 stabilization [250,251]. Wang *et al*. have reported the ability of quinacrine and other acridine derivatives to activate wild-type p53 transcription in ovarian cancer, non-small cell-lung carcinoma and colon adenocarcinoma cell lines, independent of DNA damage and MDM2 [251]. This DNA damage- and MDM2-independent effect of quinacrine in activating wild-type p53 in a diverse set of cell lines was supported by Gurova *et al*. when they found that quinacrine activates p53 in renal cell carcinomas, non-small cell lung carcinoma, colon and breast carcinomas, prostate adenocarcinomas, and fibrosarcomas [250]. Friedman et al. extended these findings to reveal that quinacrine activates p53 in several different head and neck squamous cell carcinoma cell lines with wild-type p53 [249]. The cell death induced after quinacrine treatment was not only p53 dependent [250], but also involved Bcl-2-associated X protein (BAX) [251], thereby indicating an important role of the mitochondrial apoptosis pathway. This suggests that other signaling proteins may also be involved in the cell death induction by quinacrine. The mechanism of p53 activation by quinacrine and its ability to modulate other signaling proteins may minimize the toxic side effects seen with treatments using DNA-binding platinum agents, making it potentially a desirable anticancer agent.

## Quinacrine and NF-κB

The unique mechanism of p53 upregulation which differs from the genotoxic upregulation of p53 was not investigated by Wang *et al*. [251], but Gurova *et al*. [250], found the induction of p53 to be linked to the inhibition of NF-κB. These results were later extended using a skin inflammation mouse model where the contact hypersensitivity (CHS) response to chemical allergen sensitization was evaluated [253]. In this mouse model the authors identified NF-κB to be critical in the development of the contact hypersensitivity response and demonstrated that quinacrine reduced CHS by inhibiting NF-κB activation and as well as cytokines (TNFα, IL-1β, and CCL21) that are dependent on NF-κB activation [253].

NF-κB is also a key regulator of cytokine-induced expression of endothelial cellular adhesion molecules (CAMs) [254,255]. The inhibitory effect of quinacrine on NF-κB in this context was supported in experiments where NiCl_2_- and CoCl_2_-induced cellular activation of ICAM-1 was inhibited by quinacrine [256]. The effect was attributed to PLA_2 _because the enzyme causes the generation of platelet activation factor and eicosanoids [257], which are thought to play a role in the activation of NF-κB [256].

NF-κB has been recently shown to also depend on arachidonic acid metabolites [258] and upstream inhibition by quinacrine has been proposed to inhibit the activation of NF-κB due to its inhibitory effects on PLA_2 _[259]. In a study evaluating the effect of lysophosphatidic acid (LPA) on endothelial cell activation, quinacrine blocked LPA-stimulated activation of NF-κB as well as the increase in expression of genes known to be dependent on the activation of the NF-κB transcription factor: E-selectin, ICAM-1, IL-8, and MCP-1 [260].

Another interesting line of investigation further revealed that reactive oxygen intermediates (ROI) are implicated in UVB-induced expression of TNFα in keratinocytes and that COX products and, more importantly, LOX products, also known as eicosanoids, which are themselves products of an oxidative metabolism, are the main ROI implicated in this induction [261]. The investigators hypothesize that eicosanoids likely exert their function through activation of NF-κB [261]. They also attributed the reduction of TNFα mRNA after quinacrine administration to the inhibitory activity of quinacrine on PLA_2_, based on reports showing that UVB can induce PLA_2 _in keratinocytes [262-264]. Another study investigating the source of oxygen radicals which activate Kupffer cell NF-κB after co-culture with AH70 cells attributed the attenuation of oxidative NF-κB activation to the PLA_2 _inhibitory activity of quinacrine [265].

However, because many studies do not directly implicate PLA_2 _inhibition by quinacrine as the mechanism of NF-κB inhibition and in light of more recent investigations challenging the notion that quinacrine acts primarily as an inhibitor of PLA_2 _[200,253,266], quinacrine's effect is presumably, at least partially, due to NF-κB inactivation via a mechanism other than PLA_2 _inhibition. Pupe et al. [261] present another intriguing mechanism for NF-κB inactivation as their experiments revealed quinacrine to inhibit UVB-induced IκBα degradation. However, this type of inhibition may be tumor-specific since another mechanism of NF-κB inhibition, nuclear translocation and sequestration of an inactive complex, has been well documented.

Unlike other NF-κB inhibitors, quinacrine does not inhibit the NF-κB pathway via cytoplasmic sequestration of p65. Instead, published experiments indicate a mechanism involving confinement of the p65 complex in the nucleus in an inactive state [250]. The increased presence of NF-κB in the nucleus after quinacrine exposure was supported by experiments revealing increased DNA binding of NF-κB after quinacrine treatment alone or in combination with TNFα [267]. These experiments also revealed that quinacrine plus TNFα induced greater NF-κB DNA binding than TNFα treatment alone. The lack of DNA binding inhibition of NF-κB after quinacrine treatment was also confirmed in a report by Fabbri *et al*. [268].

Down-regulation of p65 Ser536 phosphorylation by IKKα has been suggested as the primary mechanism of action for quinacrine [250]. The physiological importance of this inhibition was recently confirmed in the skin inflammation mouse model described in a previous section [253]. Further support of this mechanism was attained by Na *et al*. when hydrogen peroxide-induced phosphorylation of the p65 subunit of NF-κB was partially inhibited by quinacrine [269]. The effects of quinacrine on NF-κB are in line with its uses in the treatment of inflammatory diseases as a single agent or in combination with other medications [21]. As discussed further below, first hints of the mechanism by which quinacrine may inhibit the NF-κB pathway and promote the p53 pathway have come from studies with 9-aminoacridine (9AA), which implicated AKT and mTOR as targets for quinacrine [270].

## Quinacrine and AKT

As shown in Figure 3, AKT is involved in the NF-κB and p53 signaling pathways [245,271,272]. AKT phosphorylates the p65 subunit of NF-κB at Ser536, ultimately stimulating NF-κB transcriptional activity [272,273]. In addition, AKT phosphorylates MDM2 on Ser166 [271]. AKT phosphorylation of MDM2 induces translocation of MDM2 into the nucleus and targets p53 for destruction [271]. Phosphorylated MDM2 also transports p53 from the nucleus to the cytoplasm where it is involved in the induction of p53 degradation through the proteasome. Therefore, AKT is a critical signaling protein involved in the suppression of p53 activity. This hypothesis has been supported by experiments demonstrating a correlation between AKT kinase activity and inhibition of p53 [272].

Guo *et al*. demonstrated that 9AA inhibits AKT activity and its phosphorylation at Ser473 [270]. They went on to show that this inhibition was not a direct effect of reduced PI3K activity and implicated mTOR in this inhibition. Hence, it seems that acridines like quinacrine may be involved in stopping a positive feedback loop between AKT and mTOR [270]. The inhibition of AKT activity by 9AA has also been confirmed by other investigators in a model of human T-cell leukemia virus-transformed cells [274]. Furthermore, in a study of the role of arachidonic acid metabolism and epidermal growth factor (EGF) receptor in neurotensin-induced prostate cancer cell growth, quinacrine's activity as an inhibitor of AKT was reaffirmed. These experiments revealed quinacrine to inhibit neurotensin-, and to a lesser degree EGF-stimulated phosphorylation of AKT [275].

## The multiple actions of quinacrine and its established history of safety make it an attractive anti-neoplastic chemotherapeutic agent

Since its discovery as a potent antimalarial compound, quinacrine has been effective not only in the treatment, but also as a prophylaxis for malaria as well as a medication for a wide range of other disorders. Due to its anti-inflammatory activity in patients with autoimmune disorders quinacrine has been used to treat lupus erythematosus, rheumatoid arthritis, bronchial asthma and other inflammatory diseases. The safety of and bioavailability of quinacrine has been demonstrated as patients with these diseases used quinacrine for months at a time to control their symptoms. The pharmacokinetics and safety of quinacrine has been extensively studied as it was administered as a protective measure to millions of US soldiers in the Pacific region during World War II.

Some of the more serious side effects of quinacrine are mild in comparison to other anti-cancer chemotherapeutics and most of the conditions can be easily reversed after treatment cessation or dose reduction. Many of quinacrine's side effects develop gradually, starting from minor lesions in the case dermatitis or a slight decrease in blood counts in the development of anemia, and have been found to be completely and easily reversible, if quinacrine use is discontinued at this early stage [21,49,64,276]. Indeed some of the side effects exhibited due to quinacrine treatment can be used in the clinical setting to confirm proper dosing of the drug in the treatment of cancer patients. The yellow discoloration of the skin due to the accumulation of the bright yellow compound would indicate to the clinician that the drug has reached the equilibrium and as in the case of squamous cell carcinomas, has potentially reached areas where tumor has developed.

Furthermore, the polypharmacology of quinacrine make it an attractive drug in the use of different cancer types. In addition, as inflammation is now being considered the seventh hallmark of cancer [277], quinacrine's anti-inflammatory effects would seem to increase its potential utility as a anti-cancer drug. As more research is being conducted into quinacrine's mechanisms of action, investigators have begun to realize that its interactions extend beyond mere DNA binding and effects on nuclear proteins. Quinacrine has thus been shown to bind and inhibit proteins involved in multidrug resistance, to disrupt the arachidonic acid pathway, as well as affecting the p53, NF-κB and AKT pathway. Its effects on multiple key signaling pathways, implicated in the malignant progression of numerous cancer types, make quinacrine an exciting candidate as a chemotherapeutic agent in new types of combination treatments. Continued research into the mechanisms of this drug is clearly warranted as it may be used in addition to established therapeutic regimes in hopes of ultimately reducing toxic side effects of drugs, such as DNA damaging agents, currently used in the clinic.

## Competing interests

The authors declare that they have no competing interests.

## Authors' contributions

All authors contributed to the writing of this manuscript and approved the final version.
